# Resveratrol Induces Growth Arrest and Apoptosis through Activation of FOXO Transcription Factors in Prostate Cancer Cells

**DOI:** 10.1371/journal.pone.0015288

**Published:** 2010-12-14

**Authors:** Qinghe Chen, Suthakar Ganapathy, Karan P. Singh, Sharmila Shankar, Rakesh K. Srivastava

**Affiliations:** 1 Virginia Bioinformatics Institute, Virginia Polytechnic Institute and State University, Blacksburg, Virginia, United States of America; 2 Division of Radiation Biology, Department of Radiation Oncology, The University of Texas Health Science Center at San Antonio, San Antonio, Texas, United States of America; 3 Department of Biostatistics, University of North Texas Health Science Center at Fort Worth, Fort Worth, Texas, United States of America; 4 Department of Pathology and Laboratory Medicine, The University of Kansas Cancer Center, The University of Kansas Medical Center, Kansas City, Kansas, United States of America; 5 Department of Pharmacology, Toxicology and Therapeutics, and Medicine, The University of Kansas Cancer Center, The University of Kansas Medical Center, Kansas City, Kansas, United States of America; Enzo Life Biosciences, United States of America

## Abstract

**Background:**

Resveratrol, a naturally occurring phytopolyphenol compound, has attracted extensive interest in recent years because of its diverse pharmacological characteristics. Although resveratrol possesses chemopreventive properties against several cancers, the molecular mechanisms by which it inhibits cell growth and induces apoptosis have not been clearly understood. The present study was carried out to examine whether PI3K/AKT/FOXO pathway mediates the biological effects of resveratrol.

**Methodology/Principal Findings:**

Resveratrol inhibited the phosphorylation of PI3K, AKT and mTOR. Resveratrol, PI3K inhibitors (LY294002 and Wortmannin) and AKT inhibitor alone slightly induced apoptosis in LNCaP cells. These inhibitors further enhanced the apoptosis-inducing potential of resveratrol. Overexpression of wild-type PTEN slightly induced apoptosis. Wild type PTEN and PTEN-G129E enhanced resveratrol-induced apoptosis, whereas PTEN-G129R had no effect on proapoptotic effects of resveratrol. Furthermore, apoptosis-inducing potential of resveratrol was enhanced by dominant negative AKT, and inhibited by wild-type AKT and constitutively active AKT. Resveratrol has no effect on the expression of FKHR, FKHRL1 and AFX genes. The inhibition of FOXO phosphorylation by resveratrol resulted in its nuclear translocation, DNA binding and transcriptional activity. The inhibition of PI3K/AKT pathway induced FOXO transcriptional activity resulting in induction of Bim, TRAIL, p27^/KIP1^, DR4 and DR5, and inhibition of cyclin D1. Similarly, resveratrol-induced FOXO transcriptional activity was further enhanced when activation of PI3K/AKT pathway was blocked. Over-expression of phosphorylation deficient mutants of FOXO proteins (FOXO1-TM, FOXO3A-TM and FOXO4-TM) induced FOXO transcriptional activity, which was further enhanced by resveratrol. Inhibition of FOXO transcription factors by shRNA blocked resveratrol-induced upregulation of Bim, TRAIL, DR4, DR5, p27^/KIP1^ and apoptosis, and inhibition of cyclin D1 by resveratrol.

**Conclusion/Significance:**

These data suggest that FOXO transcription factors mediate anti-proliferative and pro-apoptotic effects of resveratrol, in part due to activation of extrinsic apoptosis pathway.

## Introduction

Prostate cancer, one of the more common neoplasms in the western world, arises through the progressive development of one or more pre neoplastic lesions into adenocarcinoma, and subsequently to metastatic disease [1]. Recent advances have identified key genetic alterations that can initiate prostate carcinogenesis, and enhance the probability of cancer progression. Prostate cancer cells are only modestly responsive or even unresponsive to the cytotoxic effects of chemotherapeutic agents or radiotherapy. Increased concentrations of cytotoxic drugs and higher dosages of irradiation fail to improve the response to therapy and it leads to resistance to apoptosis in prostate cancer cells. Thus, it is imperative to identify anticancer agents that are nontoxic and highly effective in inducing apoptosis preferentially in tumor cells.

Epidemiological data support the concept that naturally occurring compounds in the human diet may be devoid of toxicity and have long lasting beneficial effects on human health. Resveratrol has been shown to exhibit several potential chemoprotective activities in cell and animal models [2], [3], [4], [5], [6], [7], [8], [9], including inhibition of PI3K/AKT pathway [2], [6], [8], [10]. We have recently demonstrated that resveratol enhances therapeutic potential of TRAIL by upregulating death receptors (TRAIL/DR4 and TRAIL-R2/DR5) and also engaging mitochondrial pathway of apoptosis [2], [3]. Deletion or mutation of PTEN has been the main cause for constitutively active AKT leading to prostate carcinogenesis in humans [11], [12]. Thus, resveratrol may be potential candidate to target cells with PTEN deletion or inactivation and enhanced AKT activity in precancerous prostate tissue.

PI3K signaling plays a pivotal role in intracellular signal transduction pathways involved in cellular transformation, cell growth, and tumorigenesis. Inactivation of AKT results in dephosphorylation and activation of FOXO transcription factors, reported to mediate cell cycle arrest, DNA repair, and apoptosis [13], [14]. These transcription factors, belong to the ‘O’ subgroup of winged-helix/forkhead transcription-factor family, consist principally of four members FOXO1, FOXO3a, FOXO4, and FOXO6 [15]. FOXO proteins are evolutionarily conserved transcription factors implicated in several fundamental cellular processes, functioning as end-point for transcriptional programs involved in apoptosis, stress response and longevity [16], [17], [18], [19], [20]. Abrogation of FOXO function is frequently observed in human cancer [17], [21], therefore the mechanisms of regulation of the FOXO proteins are receiving increasing attention in cancer research. The FOXO proteins integrate regulatory inputs from a variety of upstream signaling pathways, most importantly in response to growth factor and stress signaling [17], [21]. Recently, FOXO factors have been established as tumor suppressors, promoting the transcription of pro-apoptotic molecules like FasL and Bim when the PI3K/AKT pathway is downregulated due to nutrient or serum starvation and cellular stress [22], [23], [24]. Triple knockout mouse models proved the tumor suppressor properties of FOXOs, as mice simultaneously lacking the principal members of the mammalian FOXO subfamily, FOXO1, FOXO3a and FOXO4, are prone to develop hemangiomas and lymphoproliferative diseases [25]. Conversely, the individual or paired inactivation of FOXO1, FOXO3a or FOXO4 resulted in a less severe phenotype, supporting the idea of functional redundancy of these FOXO factors [25]. Furthermore, forced expression of FOXO has been shown to inhibit tumorigenesis in xenograft models in nude mice [26], [27], [28], [29], [30], [31], [32]. Therefore, reactivation of FOXO based on its tumor suppressor properties is considered as a very attractive anti-cancer strategy. Since FOXO proteins were reported to be critical mediators of apoptosis induced by anticancer drugs, we postulated that FOXO expression or transcriptional activity could be important event in mediating the effects of resveratrol.

The objectives of our study were to examine the effects PI3K/AKT pathway on the regulation of FOXO transcription factors and their target gene products, and assess whether PI3K/AKT/FOXO pathway regulates anti-proliferative effects of resveratrol in prostate cancer cells. Our data demonstrated that inhibition of PI3K/AKT pathway enhanced FOXO DNA-binding and transcriptional activity, resulting in the regulation of its gene products (TRAIL, DR4, DR5, Bim, p27^/KIP1^ and cyclin D1). Inhibition of PI3K/AKT pathway or overexpression of FKHR, FKHRL1 and AFX enhanced resveratrol-induced FOXO transcriptional activity. In contrast, inhibition of FKHR, FKHRL1 or AFX blocked resveratrol-induced expression of TRAIL, DR4, DR5, Bim and p27, and inhibited the expression of cyclin D1. These data suggest that resveratrol induces apoptosis and growth arrest through activation of FOXO transcription factors, and inhibition of PI3K/AKT pathway activates FOXO transcription factors and further enhances antiproliferative and proapoptotic effects of resveratrol.

## Results

### Inhibition of PI3K/AKT pathway enhances resveratrol-induced apoptosis in prostate cancer cells

**Figure 1:**
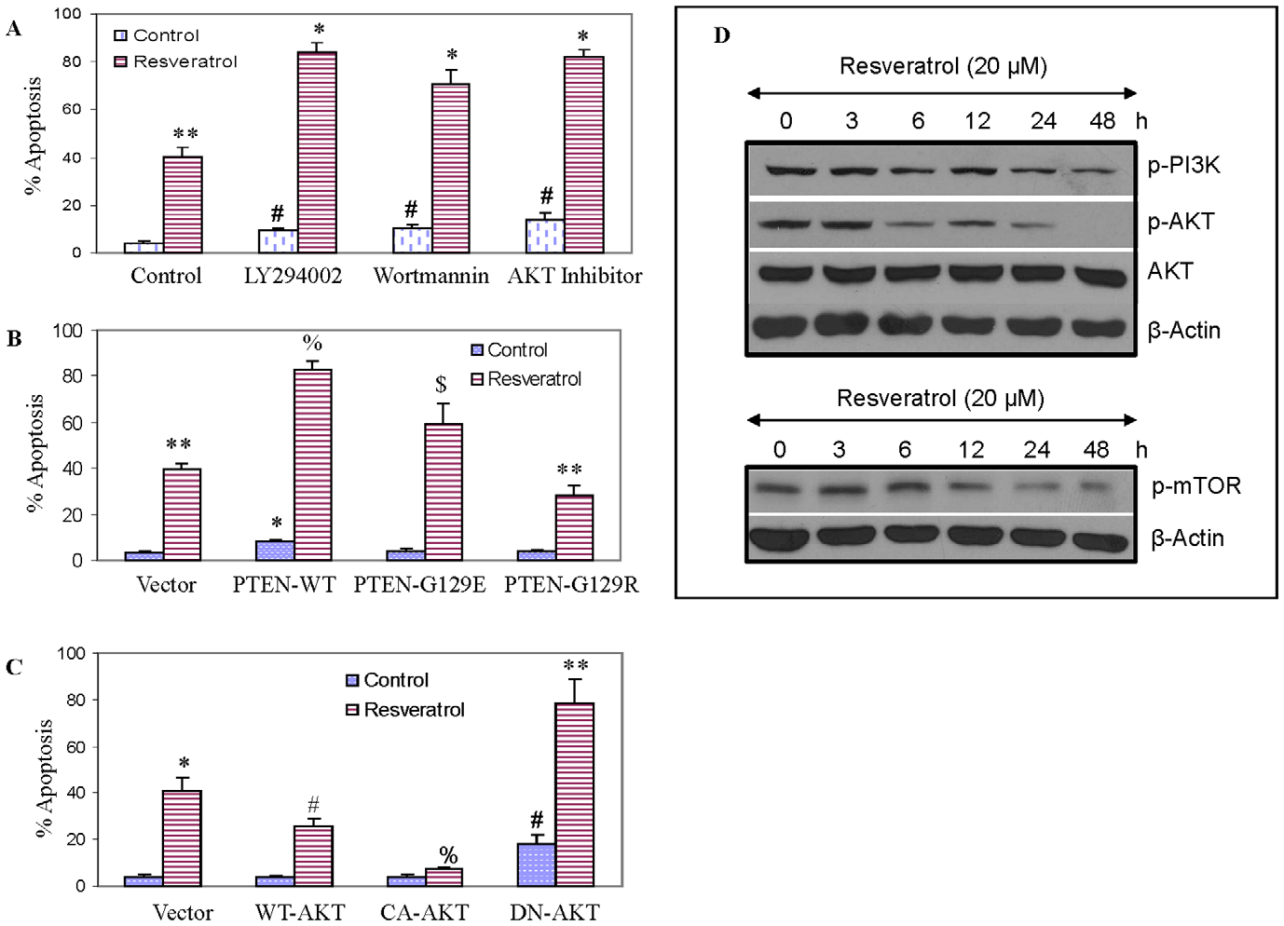
Inhibition of PI3K/AKT pathway enhances apoptosis-inducing potential of resveratrol. (A), LNCaP cells were pretreated with LY294002 (1 µM), Wortmannin (1 µM) or AKT inhibitor (100 nM) for 2 h, followed by treatment with resveratrol (20 µM) for 48 h. At the end of incubation period, cells were harvested and apoptosis was measured by TUNEL assay. Data represent mean ± SE. *, # and **  =  significantly different from respective control (P<0.05). (B), LNCaP cells were transiently transfected with empty vector, PTEN-WT, PTEN-G129E or PTEN-G129R. The culture medium was changed and cells were treated with or without resveratrol (20 µM) for 48 h, and apoptosis was measured by TUNEL assay. Data represent mean ± SE. *, **, % and $  =  significantly different from respective control (P<0.05). (C), LNCaP cells were transiently transfected with empty vector, WT-AKT, CA-AKT or DN-AKT. The culture medium was changed and cells were treated with or without resveratrol (20 µM) for 48 h, and apoptosis was measured by TUNEL assay. Data represent mean ± SE. *, **, # and %  =  significantly different from respective control (P<0.05). (D), Effects of resveratrol on the expression of PI3K, AKT and mTOR pathway. LNCaP cells were treated with resveratrol (20 µM) for various time points. Western blots were performed with anti-phospho-PI3K, phospho-AKT, anti-AKT and phospho-mTOR antibodies. β-Actin was used as a loading control. The data are representative of three experiments.

We first examined the effects of pharmacological inhibition of AKT by LY294002, Wortmannin or AKT inhibitor on resveratrol-induced apoptosis in LNCaP cells (Fig. 1A). Resveratrol induced apoptosis in LNCaP cells. Similarly, LY294002, Wortmannin or AKT inhibitor slightly but significantly induced apoptosis. Pretreatment of LNCaP cells with LY294002, Wortmannin or AKT inhibitor significantly enhanced resveratrol-induced apoptosis at 48 h. These data suggest that inhibition of PI3K/AKT activity by pharmacological approach enhanced resveratrol-induced apoptosis.

The tumor suppressor gene PTEN is quite often inactivated in human prostate cancers. The inactivation of PTEN can cause constitutive phosphorylation and activation of AKT, leading to enhanced cell growth and tumor progression. Since LNCaP cells express constitutively active AKT, we sought to examine the role of PI3K/AKT pathway on resveratrol-induced apoptosis. LNCaP cells were transfected with empty vector or plasmid expressing wild type PTEN, PTEN-G129E, or PTEN-G129R and incubated in the presence or absence of resveratrol (Fig. 1B). Resveratrol induced apoptosis in LNCaP cells transfected with empty vector. Transfection of LNCaP cells with wild type PTEN significantly enhanced resveratrol-induced apoptosis. Overexpression of PTEN-G129E or PTEN-G129R in LNCaP cells significantly inhibited resveratrol induced apoptosis than those transfected with wild type PTEN. These data suggest that inhibition of AKT activity by overexpression of PTEN enhances apoptosis-inducing potential of resveratrol.

Since overexpression of PTEN enhances resveratrol-induced apoptosis, we regulated AKT expression by wild type AKT (WT-AKT), constitutively active AKT (CA-AKT) and dominant negative AKT (DN-AKT). LNCaP cells were transiently transfected with empty vector, WT-AKT, CA-AKT or DN-AKT and treated with or without resveratrol (20 µM). Transfection of LNCaP cells with empty vector, WT-AKT or CA-AKT had no effect on apoptosis (Fig. 1C). Resveratrol induced apoptosis in empty vector transfected cells. Overexpression of WT-AKT or CA-AKT in LNCaP cells inhibited resveratrol-induced apoptosis. Overexpression of DN-AKT alone significantly induced apoptosis. Interestingly, overexpression of DN-AKT enhanced resveratrol-induced apoptosis. Overall, these data suggest that inhibition of PI3K/AKT pathway by genetic and pharmacological means enhance resveratrol-induced apoptosis in prostate cancer cells.

### Resveratrol inhibits phosphorylation of PI3K and AKT

Since PI3K/AKT pathway inhibits resveratrol-induced apoptosis, we sought to examine the effects of resveratrol on the phosphorylation of PI3K and AKT (Fig. 1D). LNCaP cells were treated with resveratrol and phosphorylation of PI3K and AKT was examined by the Western blot analysis. Resveratrol inhibited the phosphorylation of PI3K and AKT in a time dependent manner. The phosphorylation of these kinases was abolished at 48 h. Resveratrol had no effect on the expression of total AKT.

Since mTOR is a down-stream of AKT, we next sought to examine whether resveratrol also regulates phosphorylation of mTOR. As shown in Fig. 1D, resveratrol inhibited the phosphorylation of mTOR. These data suggest that resveratrol can inhibit the phosphorylation of both PI3K/AKT and mTOR proteins, and thus play a major role in mediating anti-apoptotic effects of resveratrol.

### Resveratrol has no effect on the expression of FKHR, FKHRL1 and AFX, but inhibits the phosphorylation of FOXO proteins

**Figure 2:**
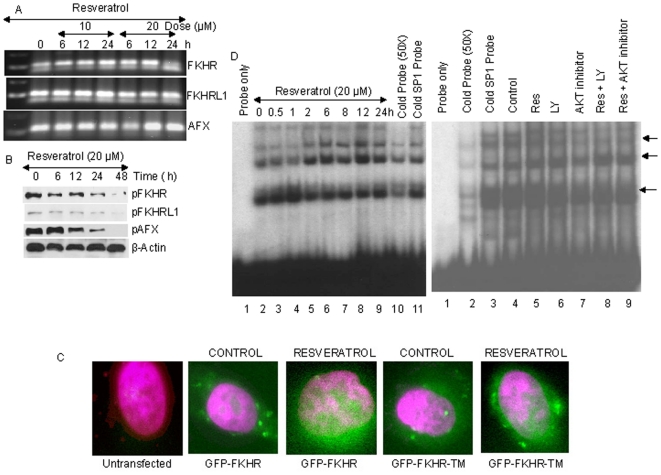
Regulation of FOXO by resveratrol. (A), Effects of resveratrol on the expression of FKHR, FKHRL1 and AFX. LNCaP cells were treated with resveratrol (10 or 20 µM) for 6, 12 or 24 h. Total RNA was isolated and RT-PCR analysis was performed to measure the expression of FKHR, FKHRL1 and AFX. (B), Effects of resveratrol on the phosphorylation of FOXO proteins. LNCaP cells were treated with resveratrol (20 µM) for 0–48 h, and the Western blot analyses were performed to measure the expression of phospho-FKHR, phospho-FKHRL1 and phosphor-AFX. β-Actin was used as a loading control. (C), Resveratrol induces translocation of FKHR to nucleus. LNCaP cells were seeded in chambered slides and transfected with GFP-FKHR or GFP-FKHR-TM (phosphorylation deficient triple mutant). The culture medium was changed and cells were treated with or without resveratrol (20 µM) for 24 h. Cells were fixed, permeabilized, and stained with DAPI and visualized under a fluorescence microscope. green color  =  FKHR, red color  =  nucleus (for clarity the color of nucleus was changed from blue to red), yellow  =  colocalization of FKHR to nucleus. (D), Left panel, LNCaP cells were treated with resveratrol (20 µM) for various time points (0–24 h). Nuclear extracts were prepared and the gelshift experiment was performed as described in Materials and Methods. Lane 1 =  probe only, lanes 2–9 =  resveratrol treated samples, lane 10 =  cold probe (50×), and lane 11 =  cold SP1 probe. The data are representative of three experiments. Right panel, LNCaP cells were untreated or treated with resveratrol (20 µM) in the presence or absence of LY (1 µM) or AKT inhibitor (100 nM) for 24 h. Nuclear extracts were prepared and the gelshift experiment was performed as described in Materials and Methods. Lane 1 =  probe only, lane 2 =  cold probe (50×), lane 3 =  cold SP1, lane 4 =  control, lane 5 =  resveratrol, lane 6 =  LY, lane 7 =  AKT inhibitor, lane 8 =  resveratrol plus LY, lane 9 =  resveratrol + AKT inhibitor. The data are representative of three experiments.

PI3K/AKT pathway has been shown to regulate the phosphorylation of FOXO proteins [33]. We therefore measured the expression of FOXO genes by RT-PCR, and phosphorylation of FOXO proteins by Western blot analysis. Resveratrol had no effect on the expression of FKHR, FKHRL1 and AFX as measured by RT-PCR (Fig. 2A). We next sought to examine whether resveratrol regulates phosphorylation of FOXO proteins. As shown in Fig. 2B, resveratrol inhibited the phosphorylation of FKHR, FKHRL1 and AFX. These data suggest that resveratrol can inhibit the phosphorylation of FOXO proteins, which are down stream of AKT, and dephosphorylation of FOXO by resveratrol may result in its activation through nuclear translocation.

### Resveratrol enhances translocation of wild type and mutant FKHR to nucleus and enhances FOXO-DNA interaction

We next examined the nuclear translocation of FKHR by resveratrol using wild type and mutant GFP-FKHR constructs. LNCaP cells were transfected with either GFP-FKHR or GFP-FKHR-TM (triple mutant) and treated with resveratrol for 24 h (Fig. 2C). Translocation of wild type and phosphorylation deficient mutant FKHR was observed by fluorescence microscopy. Resveratrol induced the nuclear translocation of both wild type and mutant FKHR. However, the nuclear translocation of FKHR-TM was much higher than wild type FKHR.

We next examined the FOXO-DNA interaction by electrophoretic mobility shift assay (EMSA). An electrophoretic mobility shift assay (EMSA) is a common affinity electrophoresis technique used to study protein-DNA interaction. Treatment of LNCaP cells with resveratrol resulted in enhanced FOXO-DNA interaction (Fig. 2D, left panel). The incubation of cold probe with the nuclear extract resulted in reduced FOXO-DNA binding activity. By comparison, the incubation of non-specific cold SP1 probe had no effect on FOXO-DNA binding. The combination of LY294002 or AKT inhibitor with resveratrol slightly enhanced the FOXO-DNA binding activity, than single agent alone (Fig. 2D, right panel). Overall, these data suggest that resveratrol can enhance FOXO nuclear translocation and DNA binding activity.

### Inhibition of PI3K/AKT pathway and phosphorylation deficient mutants of FOXO proteins enhance resveratrol-induced FOXO transcriptional activity

**Figure 3:**
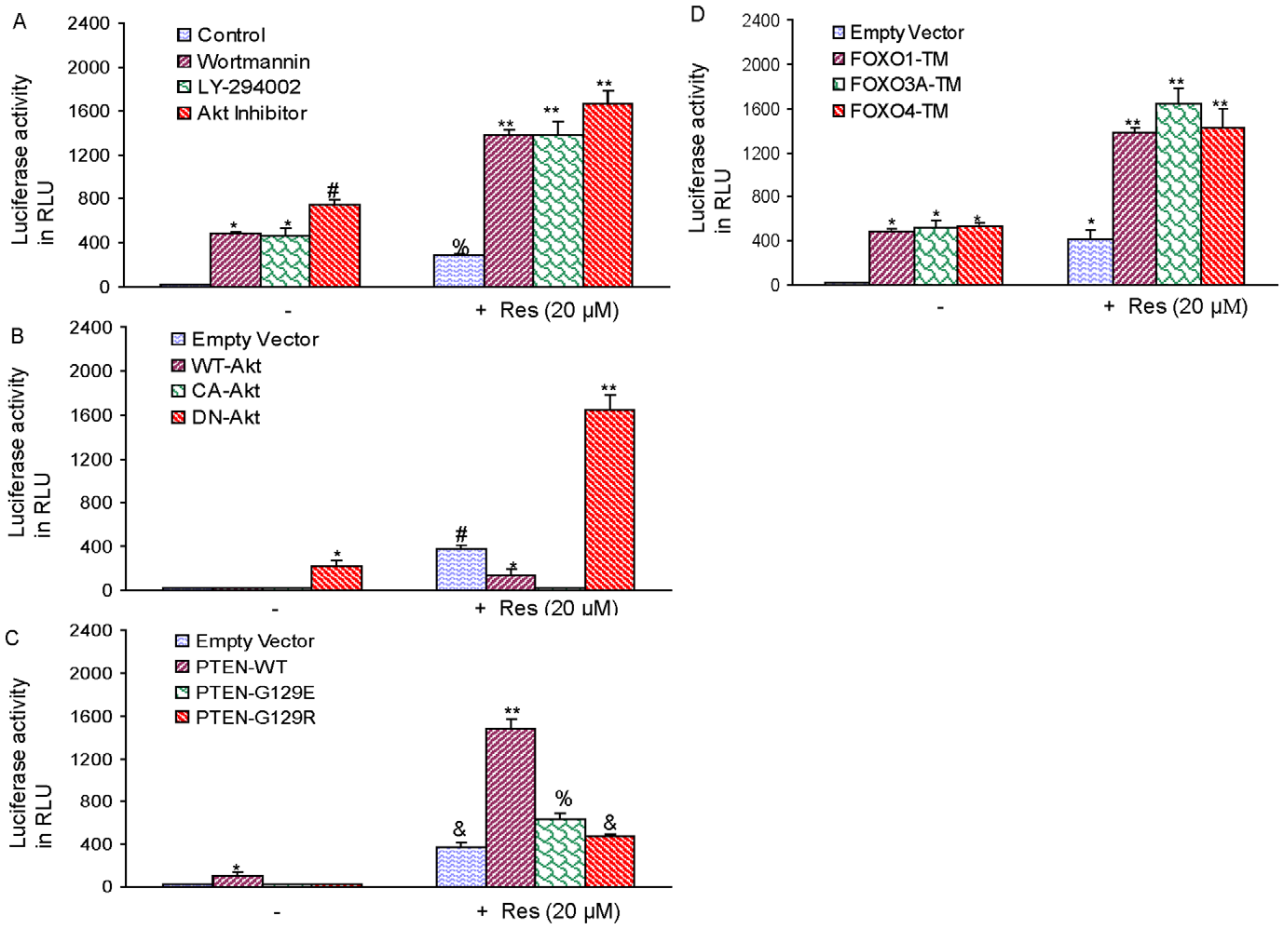
Inhibition of PI3K/AKT pathway or overexpression of phosphorylation deficient mutants of FOXO enhanced resveratrol-induced FOXO transcriptional activity. (A), Pharmacological inhibition of PI3K/AKT pathway enhanced resveratrol-induced FOXO activity in LNCaP cells. LNCaP cells were transiently transfected with 6X DBE-luciferase and pRL-TK plasmids for 24 h as we described elsewhere. After transfection, cells were pretreated with Wortmannin (10 µM), LY-294002 (10 µM) or AKT inhibitor IV (1 µM) for 2 h, followed by treatment with or without resveratrol (20 µM) for 24 h. Cells were harvested for firefly/Renilla luciferase assays using the Dual-Luciferase Reporter Assay System (Promega). Luciferase counts were normalized using *Renilla* luciferase transfection control. Data represent the mean ± S.D. *, #, **  =  significantly different from control, P<0.05. (B), Overexpression of dominant negative AKT enhanced resveratrol-induced FOXO activity. LNCaP cells were transiently transfected with 6X DBE-luciferase plus pRL-TK plasmids with WT-AKT, CA-AKT or DN-AKT for 24 h. After transfection, cells were pretreated with resveratrol (20 µM) for 24 h. Cells were harvested for firefly/Renilla luciferase assays using the Dual-Luciferase Reporter Assay System (Promega). Luciferase counts were normalized using *Renilla* luciferase transfection control. Data represent the mean ± S.D. *, # and **  =  significantly different from control, P<0.05. (C), Regulation of resveratrol-induced FOXO activity by wild type or mutant PTEN. LNCaP cells were transiently transfected with 6X DBE-luciferase plus pRL-TK plasmids with PTEN-WT, PTEN-G129E or PTEN-G129R for 24 h. After transfection, cells were pretreated with resveratrol (20 µM) for 24 h. Cells were harvested for firefly/Renilla luciferase assays using the Dual-Luciferase Reporter Assay System (Promega). Luciferase counts were normalized using *Renilla* luciferase transfection control. Data represent the mean ± S.D. *, &, $ and **  =  significantly different from control, P<0.05. (D), Phosphorylation deficient mutants of FOXO enhance resveratrol-induced FOXO transcriptional activity. LNCaP cells were transiently transfected with empty vector or constructs encoding FOXO1-TM, FOXO3a-TM, or FOXO4-TM together with 6X DBE-luciferase and pRL-TK plasmids for 24 h. After transfection, cells were washed, treated with resveratrol (20 µM) for 24 h, and harvested for firefly/Renilla luciferase assays using the Dual-Luciferase Reporter Assay System (Promega). Luciferase counts were normalized using *Renilla* luciferase transfection control. Data represent the mean ± S.D. * and **  =  significantly different from control, P<0.05.

We next examined whether inhibition of PI3K/AKT pathway enhances resveratrol-induced FOXO transcriptional activity in a luciferase reporter gene assay which measures FOXO function (Fig. 3A–C). We have first taken a pharmacological approach to inhibit PI3K/AKT activity by using Wortmannin, LY-294002, and AKT inhibitor IV. Wortmannin, LY-294002, AKT inhibitor IV and resveratrol alone induced FOXO transcriptional activity. Furthermore, the combination treatment of Wortmannin, LY-294002, or AKT inhibitor IV with resveratrol further enhanced FOXO activity. These data suggest that inhibition of PI3K/AKT pathway act synergistically with resveratrol to induce FOXO transcriptional activity.

We next examined the effects of AKT on resveratrol-induced FOXO activity in LNCaP cells (Fig. 3B). The AKT activity was regulated by wild type AKT (WT-AKT), constitutively active AKT (CA-AKT) and dominant negative AKT (DN-AKT). LNCaP cells were transiently transfected with empty vector, WT-AKT, CA-AKT or DN-AKT and treated with or without resveratrol (20 µM). Transfection of LNCaP cells with empty vector, WT-AKT, or CA-AKT had no effect on FOXO transcriptional activity. By comparison, transfection of cells with DN-AKT induced significant FOXO transcriptional activity. Resveratrol induced FOXO transcriptional activity in cells transfected with empty vector. Overexpression of WT-AKT or CA-AKT in LNCaP cells inhibited resveratrol-induced FOXO transcriptional activity. Interestingly, overexpression of DN-AKT enhanced resveratrol-induced FOXO transcriptional activity. Overall, these data suggest that inhibition of AKT activity by genetic approach enhances resveratrol-induced FOXO transcriptional activity in prostate cancer cells.

Since LNCaP cells express mutant PTEN resulting in constitutive activation of AKT, we sought to examine the role of PTEN on resveratrol-induced FOXO transcriptional activity (Fig. 3C). LNCaP cells were transfected with empty vector or plasmid expressing wild type PTEN, PTEN-G129E, or PTEN-G129R and treated with or without resveratrol. PTEN-WT slightly induced FOXO transcriptional activity. Resveratrol induced FOXO transcriptional activity in LNCaP cells transfected with empty vector. Transfection of LNCaP cells with wild type PTEN or PTEN-G129E significantly enhanced resveratrol-induced FOXO transcriptional activity. By comparison, overexpression of PTEN-G129R in LNCaP cells had FOXO transcriptional activity similar to that of resveratrol treated LNCaP/empty vector cells. These data suggest that inhibition of AKT activity by overexpression of PTEN enhances FOXO transcriptional activity.

We next examined whether resveratrol induces transcriptional activation of FOXO in the presence or absence of FOXO1-TM, FOXO3a-TM, or FOXO4-TM (phosphorylation deficient triple mutant) (Fig. 3D). LNCaP cells were transfected with p6xDBE-luciferase reporter construct in the presence or absence of plasmids expressing FOXO1-TM, FOXO3a-TM, or FOXO4-TM. After transfection, cells were treated with resveratrol for 24 h, and luciferase activity was measured. Transfection of cells with plasmids expressing FOXO1-TM, FOXO3a-TM, or FOXO4-TM induced FOXO transcriptional activity compared with the empty vector (control). Resveratrol-induced FOXO transcriptional activity was further enhanced in the presence of phosphorylation deficient mutants of FOXO1, FOXO3a, and FOXO4. These data indicate that activation of FOXO transcription factors further enhances resveratrol-induced FOXO transcriptional activity.

### Inhibition of PI3K/AKT pathway regulates Bim, p27^/Kip1^, cyclin D1, TRAIL, TRAIL-R1/DR4 and TRAIL-R2/DR5

**Figure 4:**
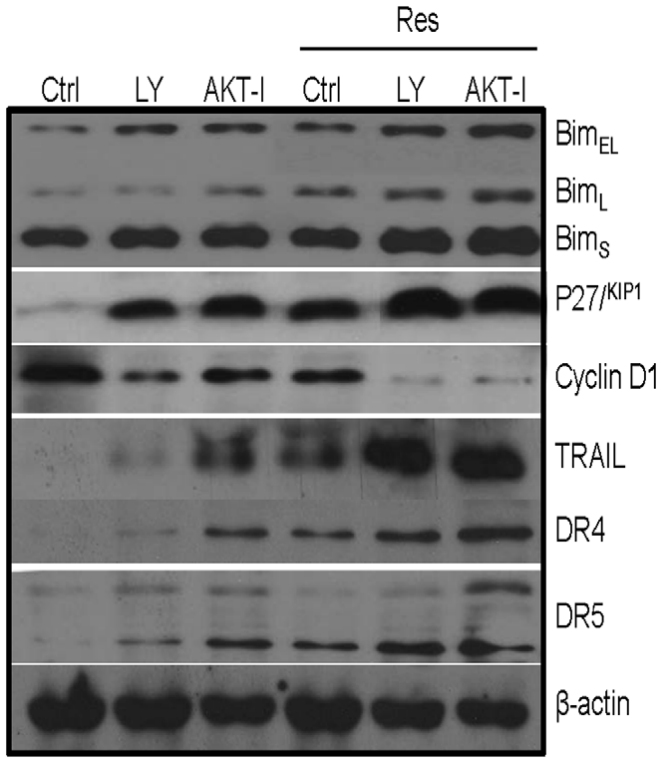
Effects of PI3K/AKT pathway and resveratrol on the expression of FOXO target genes. LNCaP cells were pretreated with LY294002 (10 µM) or AKT inhibitor (AKT-I, 1 µM) for 2 h and treated with or without resveratrol (20 µM) for 48 h. Cells were harvested to measure the expression of Bim, p27^/KIP1^, cyclin D1, TRAIL, DR4 and DR5 by theWestern blot analysis. β-actin was used as a loading control.

We next examined the effect of inhibiting PI3K/AKT pathway on the expression of Bim, p27^/Kip1^, cyclin D1, TRAIL, TRAIL-R1/DR4 and TRAIL-R2/DR5. These genes are direct targets of FOXO transcription factor. LNCaP cells were pretreated with LY-294002 or AKT inhibitor IV followed by treatment with resveratrol for 24 h, and the expression of Bim, p27, cyclin D1, TRAIL, DR4 and DR5 was measured by Western blotting (Fig. 4). LY-294002 and AKT inhibitor IV induced the expression of Bim, p27^/Kip1^, TRAIL, DR4 and DR5, and inhibited the expression of cyclin D1. Treatment of cell with resveratrol further enhanced the effects of LY-294002 or AKT inhibitor IV on the expression of Bim, TRAIL, DR4 and DR5. However, the combination of LY-294002 or AKT inhibitor IV with resveratrol had no further effect on the expression of p27^/KIP1^ and cyclin D1. These data suggest that inhibition of PI3K/AKT pathway can regulate the expression of Bim, p27^/Kip1^, cyclin D1, TRAIL, DR4 and DR5, which are transcriptional targets of FOXO. Resverarol may also regulate the expression of these FOXO transcriptional targets.

### Inhibition of FOXO expression by shRNA inhibits the antiproliferative effects of resveratrol and blocked resveratrol-induced caspase-3 activity and apoptosis

**Figure 5:**
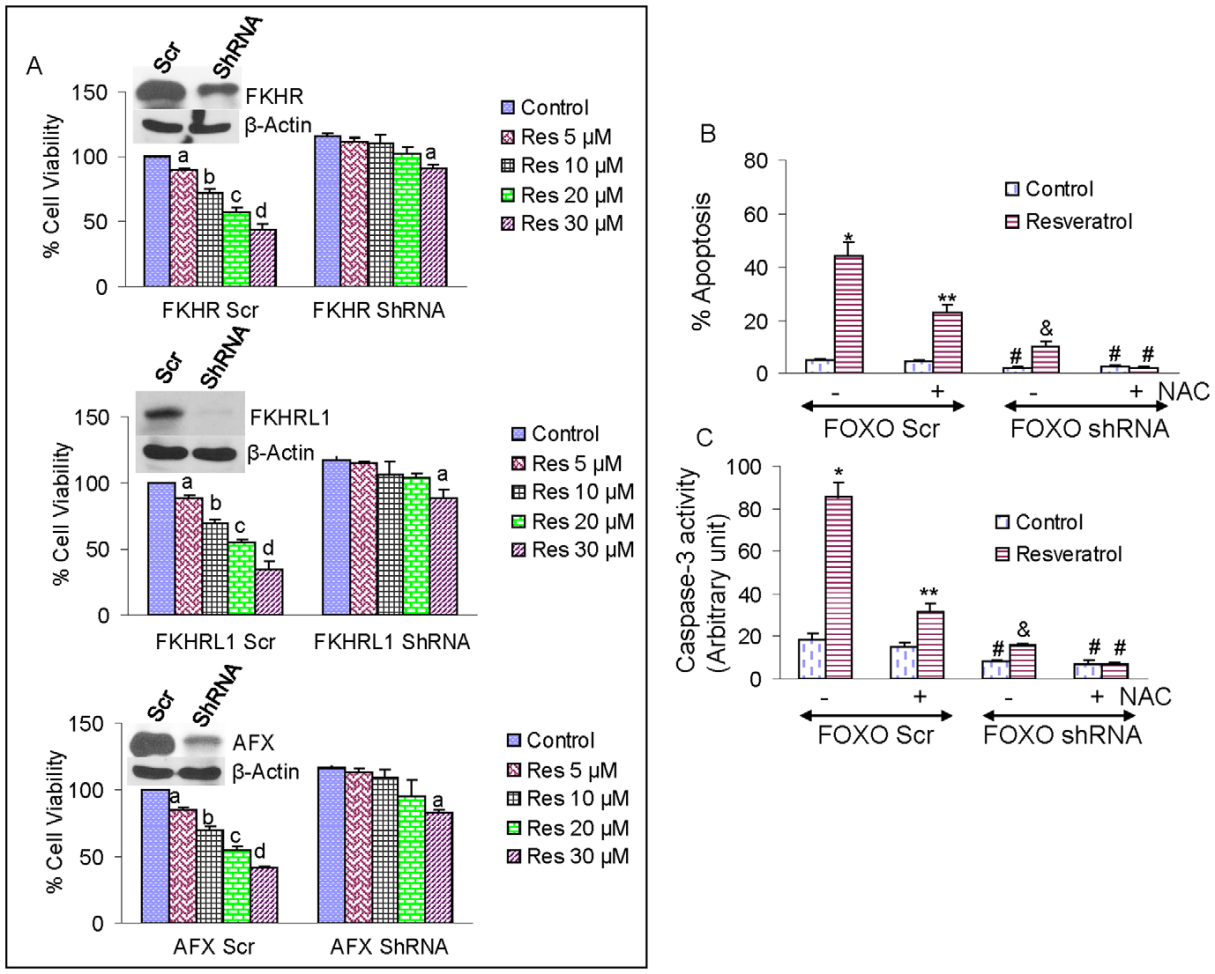
Effects of FOXO transcription factors on the regulation of antiproliferative and proapoptotic effects of resveratrol. (A), Inhibition of FOXO transcription factor by shRNA blocks anti-proliferative effects of resveratrol. LNCaP cells were transiently transfected with plasmids expressing FKHR shRNA, FKHRL1 shRNA, AFX shRNA or respective scrambled control and treated with resveratrol (20 µM) for 48 h, and cell viability was measured. (B), Inhibition of FOXO transcription factors or ROS (reactive oxygen species) by NAC blocks resveratrol-induced apoptosis. LNCaP cells were transfected with a mixture of plasmids expressing FKHR shRNA, FKHRL1 shRNA plus AFX shRNA or scrambled control. After transfection, the culture medium was changed and cells were pretreated with NAC for 2 h, and treated with resveratrol (20 µM) for 48 h. At the end of incubation period, the apoptosis was measured by TUNEL assay. (C), Inhibition of FOXO transcription factors or ROS by NAC blocks resveratrol-induced caspase-3 activity. LNCaP cells were transfected with a mixture of plasmids expressing FKHR shRNA, FKHRL1 shRNA plus AFX shRNA or scrambled control. After transfection, the culture medium was changed and cells were pretreated with NAC for 2 h, and treated with resveratrol (20 µM) for 48 h. At the end of incubation period, the caspase-3 activity was measured as per manufacturer instructions.

If resveratrol induces apoptosis by activating FOXO transcription factor, the inhibition of FOXO proteins should block the anti-proliferative effects of resveratrol. LNCaP cells were transfected with shRNA expressing FKHR, FKHRL1 or AFX and treated with resveratrol (Fig. 5). Resveratrol induced apoptosis in LNCaP/FKHR scrambled, LNCaP/FKHRL1 scrambled and LNCaP/AFX scrambled cells in a dose-dependent manner (Fig. 5A). Inhibition of FKHR, FKHRL1 or AFX by shRNA blocked anti-proliferative effects of resveratrol. These data suggest that resveratrol inhibits cell viability through regulation of FOXO transcription factors.

We next sought to examine whether inhibition of FOXO expression effect resveratrol-induced caspase-3 activity and apoptosis (Fig. 5B and C). Resveratrol induced caspase-3 activity and apoptosis in LNCaP/scrambled cells. Pretreatment of LNCaP/scrambled cells with NAC inhibited resveratrol-induced caspase-3 activity and apoptosis. Inhibition of FOXO expression by shRNA inhibited resveratrol induced caspase-3 activity and apoptosis. Interestingly, pretreatment of LNCaP/FOXO shRNA cells with NAC blocked resveratrol-induced caspase-3 activity and apoptosis. These data suggest that resveratrol induced caspase-3 activity and apoptosis through generation of ROS, and inhibition of FOXO (FKHR, FKHRL1 and AFX) inhibits caspase-3 activity and apoptosis.

### Regulation of Bim, TRAIL, DR4, DR5, p27^/Kip1^ and cyclin D1 by FOXO genes (FKHR, FKHRL1 and AFX)

**Figure 6:**
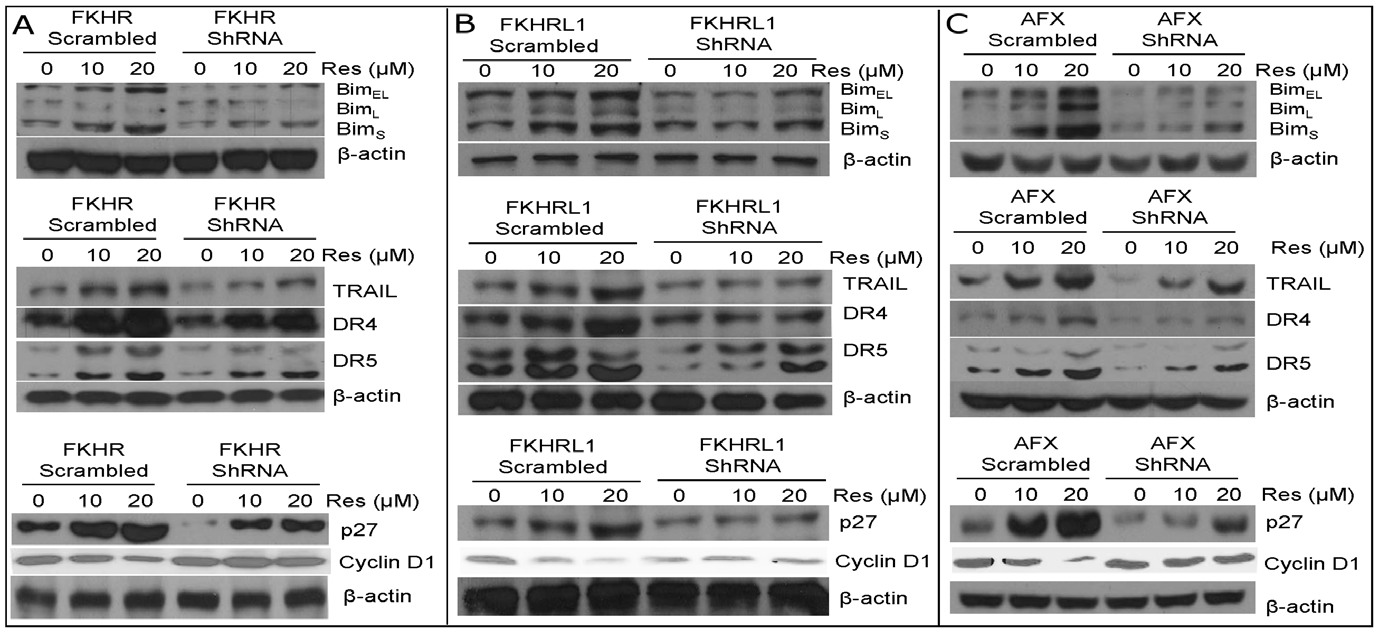
FOXO transcription factors mediate the expression of cell cycle and apoptosis-related genes. (A), LNCaP cells were transfected with plasmids expressing FKHR shRNA or scrambled control. After transfection, the culture medium was changed and cells were treated with resveratrol (0–20 µM) for 48 h. At the end of incubation period, cells were harvested to measure the expression of Bim, TRAIL, DR4, DR5, p27 and cyclin D1 by the Western blot analysis. β-actin was used as a loading control. (B), LNCaP cells were transfected with plasmids expressing FKHRL1 shRNA or scrambled control. After transfection, the culture medium was changed and cells were treated with resveratrol (0–20 µM) for 48 h. At the end of incubation period, cells were harvested to measure the expression of Bim, TRAIL, DR4, DR5, p27 and cyclin D1 by the Western blot analysis. β-actin was used as a loading control. (C), LNCaP cells were transfected with plasmids expressing AFX shRNA or scrambled control. After transfection, the culture medium was changed and cells were treated with resveratrol (0–20 µM) for 48 h. At the end of incubation period, cells were harvested to measure the expression of Bim, TRAIL, DR4, DR5, p27 and cyclin D1 by the Western blot analysis. β-actin was used as a loading control.

FOXO transcription factors have been shown to regulate apoptosis and cell cycle-related genes [14], [34]. Since resveratrol regulated the expression of Bim, TRAIL, DR4, DR5, p27^/Kip1^ and cyclin D1, we sought to examine whether these effects of resveratrol are mediated through FOXO transcription factors which were inhibited by shRNA (Fig. 6). Resveratrol induced the expression of Bim (Bim_EL_, Bim_L_ and Bim_S_), TRAIL, DR4, DR5 and p27^/Kip1^, and inhibited the expression of cyclin D1 in LNCaP/scrambled cells. Inhibition of FKHR, FKHRL1 or AFX by shRNA attenuated resveratrol-induced Bim, TRAIL, DR4, DR5 and p27^/Kip1^ expression. By comparison, inhibition of FKHR, FKHRL1 or AFX by shRNA slightly attenuated the inhibitory effects of resveratrol on cyclin D1 expression. These data suggest that resveratrol can regulate apoptosis (Bim, TRAIL, DR4, and DR5) and cell cycle-related genes (p27^/Kip1^, and cyclin D1) through FOXO transcription factors. Interestingly, induction of TRAIL and its receptors DR4 and DR5 by resveratrol will result in activation of extrinsic pathway of apoptosis.

## Discussion

In the present study, we have shown that resveratrol induced apoptosis in prostate cancer cells through inhibition of PI3K/AKT and mTOR pathway, and activation of FOXO transcription factors. Furthermore, downregulation of PI3K/AKT pathway increased resveratrol-induced apoptosis. Inhibition of FOXO transcription factors by shRNA blocked resveratrol-induced upregulation of Bim, TRAIL, DR4, DR5, p27^/Kip1^ and apoptosis, and resveratrol-induced inhibition of cyclin D1. Our data suggest that resveratrol induces cell cycle arrest and apoptosis through regulation of FOXO transcription factors in prostate cancer cells. Overall, these properties of resveratrol strongly suggest that it could be used as a cancer chemopreventive agent.

FOXO transcription factors are tumor suppressors that are inactivated in the majority of human cancers, owing to the overactivation of the PI3K/AKT pathway [35], [36], [37]. FOXO proteins can regulate a variety of genes that influence cell proliferation, survival, metabolism and response to stress [38]. The FOXOs are regulated by synthesis, phosphorylation, acetylation and ubiquitination at three different levels: subcellular localization, stability and transcriptional activity [39]. Upon the activation of PI3K/AKT signaling, FOXOs undergo AKT-mediated phosphorylation, which promotes binding to 14-3-3, nuclear export through CRM1 and cytoplasmic sequestration. Under stress conditions or in the absence of growth or survival factors, when the PI3K/AKT pathway is inhibited, FOXO proteins translocate to the cell nucleus, where their transcriptional functions can be executed [35]. In the present study, inhibition of PI3K/AKT pathway enhanced FOXO-DNA binding and transcriptional activity. Furthermore, phosphorylation deficient mutant of FOXO enhanced resveratrol-induced FOXO transcriptional activity and apoptosis. Post-translational modification of FOXO proteins is an important mechanism that regulates the ability of different transcription factors to activate distinct gene sets, involved in cell cycle inhibition [40], apoptosis [41], defense against oxidative stress and DNA repair [36], [42], [43]. The enhanced DNA binding activity also serves to limit the availability of FOXO proteins for phosphorylation by AKT [44]. So, rather than enhancing dephosphorylation of phospho-FOXO proteins, resveratrol may be simply inhibiting the rephosphorylation of FOXO proteins after they have been dephosphorylated. Thus, resveratrol treatment inhibits the accumulation of phospho-FOXO, either by inhibiting the phosphorylation of FOXO proteins in the nucleus, or by promoting the dephosphorylation of phosphorylated form of FOXO proteins in the cytoplasm.

ROS are thought to play multiple roles including tumor initiation, progression and maintenance, and ROS production is highly increased in cancer cells (Szatrowski and Nathan, 1991; Burdon, 1995). High levels of ROS under stress conditions have been shown to inhibit phosphorylation and acetylation of FOXO proteins, resulting in enhanced FOXO-DNA binding activity. In the cell, ROS levels can be sensed by virtue of stimulatory and inhibitory oxidative modification of cysteine residues within proteins that control various signaling cascades. Recently it was shown that cysteines in FOXO can also act as sensors of the local redox state. The cysteine-dependent redox switch can regulate ROS signaling pathway upstream of FOXO. Furthermore, FOXO can also control ROS levels by transcriptional regulation of a multilayered antioxidant system. Thus, cysteine based redox signaling to FOXO could play a role in fine-tuning the optimal cellular response to ROS to control organismal lifespan. In our study, phosphorylation deficient mutants of FOXO enhanced resveratrol-induced FOXO transcriptional activity and apoptosis. This may be because resveratrol induces ROS leading to induction of SIRT1 which in turn causes deacetylation of FOXO proteins and enhances DNA binding activity [44]. ROS-induced cell death was suppressed by co-transfection of a FOXO3a mutant that lacks the activation-domain of transcription, transactivation of pro-apoptotic genes by FOXO was necessary to cause ROS-induced apoptosis [45]. In fact, expression of several pro-apoptotic genes, such as Bim and Bcl-6 was induced in H_2_O_2_-stimulated cells, and was blocked by co-transfection of dominant-negative type FOXO3a mutant. These findings indicate that FOXO is a key regulator of ROS-induced apoptosis in mammalian cells.

FOXO transcription factors play important roles in regulation of cell cycle and apoptosis [46], [47]. In the present study, it appears that FKHR, FKHRL1 and AFX play overlapping roles in regulation of cell cycle and apoptosis-related genes. The inhibition of FKHR, FKHRL1 or AFX by shRNA attenuated resveratrol-induced expression of Bim, TRAIL, DR4, DR5 and p27^/kip1^ and apoptosis. Furthermore, the inhibition of FKHR, FKHRL1 or AFX by shRNA blocked the inhibitory effects of resveratrol on cyclin D1. Similar to our studies, FOXO silencing has been shown to decrease expression levels of Bim, TRAIL, FasL, p27^/Kip1^, which are FOXO target genes controlling cell cycl**e** and apoptosis [40], [48], [49], [50], [51], [52], [53]. We have recently demonstrated that inhibition of PI3K/AKT and MEK/ERK pathways act synergistically to regulate antiangiogenic effects of EGCG, resveratrol and sulforaphane through activation of FOXO transcription factors [10], [33], [54]. The activation of FOXO transcription factors through inhibition of these PI3K/AKT and MEK/ERK pathways may have physiological significance in management of diabetic retinopathy, psoriasis, cardiovascular diseases, rheumatoid arthritis and cancer.

In summary, our study demonstrate that dephosphorylation of FOXO may stabilize and enhance FOXO's DNA binding and transcriptional activity. Our findings demonstrate that resveratrol regulates the expression of FOXO's target genes such as Bim, TRAIL, DR4, DR5, cyclin D1 and p27^/KIP1^. In addition to regulation of FOXO through dephosphorylation, resveratrol may also regulate cell survival and/or apoptosis through global modulation of gene expression via deacetylation of FOXO transcription factor. Further studies are needed to assess the physiological significance of phosphorylation vs acetylation of FOXO proteins. A thorough understanding of the mechanisms of resveratrol may lead to discovery and development of novel therapeutic molecules for the treatment and prevention of human diseases.

## Methods

### Reagents

Antibodies against Bim, TRAIL, DR4, DR5, p27^/KIP1^, cyclin D1 and β-actin were purchased from Santa Cruz Biotechnology Inc. (Santa Cruz, CA). Enhanced chemiluminescence (ECL) Western blot detection reagents were from Amersham Life Sciences Inc. (Arlington Heights, IL). Terminal Deoxynucleotidyl Transferase Biotin-dUTP Nick End Labeling (TUNEL) assay kit and caspase-3 activity kit were purchased from EMD Biosciences (Gibbstown, NJ). Resveratrol was purchased from LKT Laboratories, Inc. (St. Paul, MN). All other chemicals used were of analytical grade and were purchased from Fisher Scientific (Suwanee, GA) and Sigma-Aldrich (St. Louis, MO).

### Cell Culture

LNCaP cells were obtained from the American Type Culture Collection (Manassas, VA) and cultured in RPMI 1640 supplemented with 10% fetal bovine serum (FBS) and 1% antibiotic-antimycotic at 37°C in a humidified atmosphere of 95% air and 5% CO_2_.

### Construction of FOXO shRNA vector

Expression plasmids for silencing of FKHR, FKHRL1 and AFX were constructed using psiRNA-DUO-GFP-Zeo vector (In vivoGen, San Diego, California).

### Transient Transfection

Cells were plated in 60-mm dishes in RPMI 1640 containing 10% FBS and 1% penicillin-streptomycin mixture at a density of 1×10^6^ cells/dish. The next day transfections were performed using LipofectAMINE (Invitrogen Life Technologies, Carlsbad, CA). Cells were transfected with expression constructs encoding wild type PTEN (pSG5L-HA-PTENwt), mutant PTEN (pSG5L-HA-PTEN-G 129E and pSGL5-HA-PTEN-G 129R), wild type-AKT (pUSE-WT-AKT), constitutively active-AKT (pUSE-CA-AKT), dominant negative-AKT (pUSE-DN-AKT), or the corresponding empty vectors (pSG5L or pUSE) in the presence of an expression vector pCMV-LacZ (Invitrogen Life Technologies) expressing β-galactosidase. For each transfection, 2 µg of DNA was diluted into 50 µl of medium without serum. After the addition of 3 µl of LipofectAMINE into 50 µl Opti-MEM medium, the transfection mixture was incubated for 10 min at room temperature. Cells were washed with serum-free medium, the transfection mixture was added, and cultures were incubated for 24 hrs in the incubator. The next day, culture medium was replaced with fresh RPMI 1640 containing 10% FBS and 1% penicillin-streptomycin mixture and resveratrol was added for desired times for the measurement of apoptosis and protein expression.

### Measurement of Apoptosis

Cells were treated with resveratrol and apoptosis was measured by TUNEL assay as per manufacturer instructions.

### Caspase-3 Assay

Cells (3×10^4^ per well) were seeded in a 96-well plate with 200 µl culture medium. Approximately 16 h later, cells were treated with various doses of resveratrol to induce apoptosis. Casapse-3 activity was measured as per manufacturer's instructions (EMD Biosciences) with a fluorometer.

### Western Blot Analysis

Cells were treated with resveratrol for various time points and lysed in the RIPA buffer containing 1 X protease inhibitor cocktail. Protein concentrations were determined using the Bio-Rad Protein Assay (Bio-Rad, Hercules, CA). Cell lysates containing 40 mg of protein were analyzed using 12.5% SDSPAGE. Transferred membranes were blocked using 5% skim milk and incubated overnight with primary antibodies followed by secondary antibodies conjugated with horseradish peroxidase at 1∶5,000 dilution in TBS-Tween 20 for 1 hour at room temperature. Membranes were developed using ECL Substrate. Protein bands were visualized on X-ray film using an enhanced chemiluminescence system.

### Immunocytochemistry

Cells were grown on fibronectin-coated coverslips (Beckton Dickinson, Bedford, MA), washed in PBS, and fixed for 15 min in 4% paraformaldehyde. Cells were permeabilized in 0.1% Triton X-100, washed and blocked in 10% normal goat serum. After blocking, cells were incubated with primary antibody (1∶100) for 18 h at 4°C, washed with PBS and incubated with fluorescently labeled secondary antibody (1∶200) along with DAPI (1 µg/ml) for 1 h at room temperature. Finally, coverslips were washed and mounted using Vectashield (Vector Laboratories, Burlington, CA). Isotype-specific negative controls were included with each staining. Stained cells were mounted and visualized under a fluorescent microscope.

### Luciferase Assay

LNCaP cells were transfected with empty vector, FOXO1-TM, FOXO3a-TM or FOXO4-TM along with reporter plasmids, p6xDBE-luc and pRL-TK [55]. The FOXO expression vectors (wild type and phosphorylation deficient mutants) and FOXO-luciferase constructs have been described elsewhere [55], [56]. After 24 h, transfection medium was replaced with culture medium and cells were treated with resveratrol (0–20 µM). After incubation of 24 h, the relative luciferase activity, i.e. firefly enzyme activity divided by that of the Renilla enzyme, was determined using Dual Luciferase Reporter Assay System (Promega, Madison, WI) according to the manufacturer's protocol.

### RT-PCR

Total RNA was extracted using the RNeasy Mini Kit (Qiagen, Valencia, CA) and first strand cDNA was synthesized with the Omniscript RT kit (Qiagen), using 1 µg of RNA per 20 µl reaction and oligo(dT) primer. cDNA was then utilized in PCR reactions for FKHR, FKHRL1, AFX and GAPDH.

### Electrophorectic mobility shift assay (EMSA)

FOXO probes were end-labeled with [γ-^32^P] dATP by incubating oligodeoxyribonucleotide strands with 5× reaction buffer and 10 U T4 polynucleotide kinase for 1 h at 37°C. Then labeled oligonucleotides were allowed to anneal at room temperature for 10 min and 20 µg protein from each sample was used in 25 µl binding reactions, which consisted of 1 µg poly dI-dC, in 5× binding buffer (50 mM Tris HCl; pH 8.0, 750 mM KCl, 2.5 mM EDTA, 0.5% Triton-X 100, 62.5% glycerol (v/v) and 1 mM DTT). To determine specificity of DNA binding, samples were incubated with or without 20 ng of unlabeled competitor DNA for 10 min at room temperature. Then 0.1 ng of labeled probe was added and samples were further incubated for 20 min at room temperature. Samples were separated on a 5% non-denaturing polyacrylamide gel in 0.5% TBE and visualized by autoradiography.

### Statistical Analysis

The mean and SD were calculated for each experimental group. Differences between groups were analyzed by one or two way ANOVA using PRISM statistical analysis software (GrafPad Software, Inc., San Diego, CA). Significant differences among groups were calculated at P<0.05.
